# MultiToxPred 1.0: a novel comprehensive tool for predicting 27 classes of protein toxins using an ensemble machine learning approach

**DOI:** 10.1186/s12859-024-05748-z

**Published:** 2024-04-12

**Authors:** Jorge F. Beltrán, Lisandra Herrera-Belén, Fernanda Parraguez-Contreras, Jorge G. Farías, Jorge Machuca-Sepúlveda, Stefania Short

**Affiliations:** 1 Department of Chemical Engineering, Faculty of Engineering and Science, Universidad de La Frontera, Ave. Francisco Salazar, 01145 Temuco, Chile; 2 Departamento de Ciencias Básicas, Facultad de Ciencias, Universidad Santo Tomas, Temuco, Chile

**Keywords:** Protein toxin, Machine learning, Prediction, Therapeutic

## Abstract

Protein toxins are defense mechanisms and adaptations found in various organisms and microorganisms, and their use in scientific research as therapeutic candidates is gaining relevance due to their effectiveness and specificity against cellular targets. However, discovering these toxins is time-consuming and expensive. In silico tools, particularly those based on machine learning and deep learning, have emerged as valuable resources to address this challenge. Existing tools primarily focus on binary classification, determining whether a protein is a toxin or not, and occasionally identifying specific types of toxins. For the first time, we propose a novel approach capable of classifying protein toxins into 27 distinct categories based on their mode of action within cells. To accomplish this, we assessed multiple machine learning techniques and found that an ensemble model incorporating the Light Gradient Boosting Machine and Quadratic Discriminant Analysis algorithms exhibited the best performance. During the tenfold cross-validation on the training dataset, our model exhibited notable metrics: 0.840 accuracy, 0.827 F1 score, 0.836 precision, 0.840 sensitivity, and 0.989 AUC. In the testing stage, using an independent dataset, the model achieved 0.846 accuracy, 0.838 F1 score, 0.847 precision, 0.849 sensitivity, and 0.991 AUC. These results present a powerful next-generation tool called MultiToxPred 1.0, accessible through a web application. We believe that MultiToxPred 1.0 has the potential to become an indispensable resource for researchers, facilitating the efficient identification of protein toxins. By leveraging this tool, scientists can accelerate their search for these toxins and advance their understanding of their therapeutic potential.

**Supplementary Information:**

The online version contains supplementary material available at 10.1186/s12859-024-05748-z.

## Introduction

Over the course of evolution, many organisms and microorganisms have developed the ability to express different types of protein toxins (PT) as part of their defense mechanisms and adaptations to the environment [1]. These proteins can be found in animals [2] and poisonous plants [3–6], as well as in pathogenic bacteria [5, 6]. PTs have a wide range of molecular targets, which has allowed them to be extensively studied as therapeutic candidates for the treatment of various diseases, generally, such as pain [7, 8], cancer [9–14], autoimmune diseases [15], cardiovascular diseases [16, 17], neurodegenerative diseases [18], viral [14, 19] and bacterial [14] infections, among others Currently, there are different proposals to classify PTs, and one of these is their classification into three main groups, (1) toxins that hinder or interfere with cellular processes through their enzymatic activity, (2) toxins that cause harm to cells by compromising the integrity of their membranes, and (3) toxins that interfere with the regular electrical functioning of the nervous system in an intoxicated organism [1]. However, the fact that PTs have a wide variety of molecular targets makes a more specific classification of these not entirely clear at present. In this regard, it has been reported that PTs can act on various molecular targets among which we find the cell membrane [20–22], voltage-gated sodium channel [23], voltage-gated calcium channel [24, 25], voltage-gated potassium channel [26, 27], acetylcholine receptor [28, 29], G-protein coupled receptor [30], and bradykinin receptor [31], among many others.

In recent years, the study of protein toxins has increased due to the great potential they represent as therapeutic drugs. In this regard, various in vitro, in vivo [32], and in silico [33] methodologies have been evaluated for their study. Among the in silico methodologies, the use of bioinformatics tools [34–37] and, more recently, machine learning (ML) [33], has gained greater relevance as it allows for the acceleration and reduction of costs of resources allocated to the search for PTs. Particularly, ML constitutes a robust and modern strategy for the discovery of pharmaceutical candidates [38, 39], with PTs being no exception in this context. Currently, there are several works based on machine learning and deep learning that generally, following a binary classification approach, allow discrimination between PTs and non-PTs. These tools are NTXpred [40], Yang and Li’s method [41], Jayaraman et al.’s method [42], Kumar et al.'s method [43], NNTox [44], TOXIFY [45], ClanTox [46], ToxClassifier [47], ToxinPred2 [33], SpiderP [48], BTXpred [49], ToxDL [50], ATSE [51], ToxIBTL [52], ToxinMI [53], Toxicity-vib [54], and CSM-Toxin [55], which have been of great utility in the field of PT study. These tools undoubtedly greatly aid in the discovery of new toxins; however, they follow a binary classification approach where the output only informs if a protein is a PT or not. Taking into account the wide variety of molecular targets that PTs act upon, it would be interesting to approach a more specific prediction method that would allow us to elucidate more specific cellular targets. Following this idea, for the first time in this work, the development of ML models for the multiple classification of 27 different classes of PTs with different modes of cellular action was evaluated.

## Methods

### Data sets

The amino acid sequences of toxins used in this work were obtained from the Universal Protein Resource (UniProt) [56]. These sequences were only selected based on the following criteria, (1) the sequence must be reviewed, (2) the sequence has at least one scientific publication demonstrating the respective PT activity, and (3) the sequence must be complete. In this regard, a certain number of PT sequences were identified considering the reported target for each of these. Below are the number of identified PT sequences (sn) with their respective cellular targets or mode of action in the cell: acetylcholine receptor inhibiting toxin (sn = 493), blood coagulation cascade activating toxin (sn = 107), blood coagulation cascade inhibiting toxin (ns = 133), bradykinin receptor impairing toxin (sn = 35), calcium-activated potassium channel impairing toxin (sn = 72), cell adhesion impairing toxin (sn = 228), chloride channel impairing toxin (sn = 20), complement system impairing toxin (sn = 27), dermonecrotic toxin (sn = 216), enterotoxin (ns = 101), fibrinogenolytic toxin (sn = 102), fibrinolytic toxin (sn = 41), G-protein coupled acetylcholine receptor impairing toxin (sn = 29), G-protein coupled receptor impairing toxin (sn = 228), hemorrhagic toxin (sn = 62), hemostasis impairing toxin (sn = 942), platelet aggregation activating toxin (sn = 74), platelet aggregation inhibiting toxin (sn = 350), potassium channel impairing toxin (sn = 664), proton-gated sodium channel impairing toxin (sn = 25), ryanodine-sensitive calcium-release channel impairing toxin (sn = 27), target cell cytoplasm (sn = 16), target cell membrane (sn = 418), voltage-gated calcium channel impairing toxin (sn = 247), voltage-gated chloride channel impairing toxin (sn = 18), voltage-gated potassium channel impairing toxin (sn = 508), and voltage-gated sodium channel impairing toxin (sn = 840) (Additional files 1–8). On the other hand, 600 random amino acid sequences of different lengths were generated, which were considered non-PT.

### Calculation of molecular descriptors and balancing of the data set

From all the sequences, the calculation of two types of molecular descriptors widely used in the development of predictive models from primary protein structures was carried out: pseudo amino acid composition (PAAC, lamda = 5, weight = 0.05) [57], and dipeptide composition descriptors (DPC) [58]. Both molecular descriptors were computed with the Python propy3 package (https://pypi.org/project/propy3/) was used for the calculation of these molecular descriptors.

Subsequently, the resulting data set was labeled for later balancing and evaluation with classification algorithms. Because the data set contains labeled classes (PT and non-PT) with an imbalanced numerical proportion, its balance was carried out through the synthetic minority over-sampling technique (SMOTE). Imbalanced data sets can cause a bias in predictive models, and in this sense, SMOTE is a data preprocessing technique used to deal with the class imbalance problem in machine learning data sets. In this technique, synthetic examples of minority classes are generated. This is done by taking examples from minority classes and creating similar but slightly modified examples, "oversampling" the minority classes to balance the data set [59]. The Python imbalanced-learn package (https://pypi.org/project/imbalanced-learn/) was used to balance the data set with SMOTE.

### Training, cross-validation, and testing

In this study, nine machine learning classification algorithms were evaluated: Random Forest (RF), Multi-layer Perceptron (MLP), eXtreme Gradient Boosting (XGBoost), Light Gradient Boosting Machine (LightGBM), Logistic Regression (LR), Naïve Bayes (NB), *k*-nearest neighbors (*k*-NN), and Quadratic Discriminant Analysis (QDA). Training with all the classifiers was conducted on 80% of the complete dataset, which underwent tenfold cross-validation. The remaining 20% of the data (independent dataset) was used to evaluate the performance of the trained models. The mentioned analyses were carried out using the libraries scikit-learn (https://pypi.org/project/scikit-learn/), XGBoost (https://pypi.org/project/xgboost/), and Microsoft LightGBM (https://pypi.org/project/lightgbm/). In this study, we evaluated the StackingClassifier, which is a meta-ensembling technique that leverages the strengths of diverse base learners by stacking their predictions as input for a final estimator. This method effectively combines multiple classification models, each of which may capture different patterns within the data. The justification for employing a StackingClassifier lies in its ability to blend various predictive models, potentially leading to better generalization on unseen data. By using predictions of base learners as features, the meta-learner can learn to correct the individual classifier mistakes, thereby improving overall accuracy. This approach is supported by empirical studies demonstrating its superiority over individual classifiers and even other ensemble methods when carefully implemented. The mean of the performance measures used to evaluate the models in cases of multiple classifications, both in the training stage through cross-validation and in the testing stage, were the following:

1 $$Sensitivity \,\left(TPR\right)=TP/(TP+FN)$$

2 $$Accuracy \,\left(ACC\right)=TP+TN/(TP+FP+FN+TN)$$

3 $$Precision\, \left(PPV\right)=TP/(TP+FP)$$

4 $$F1 \,score\, (F1)=2TP/(2TP+FP+FN)$$

**Fig. 1:**
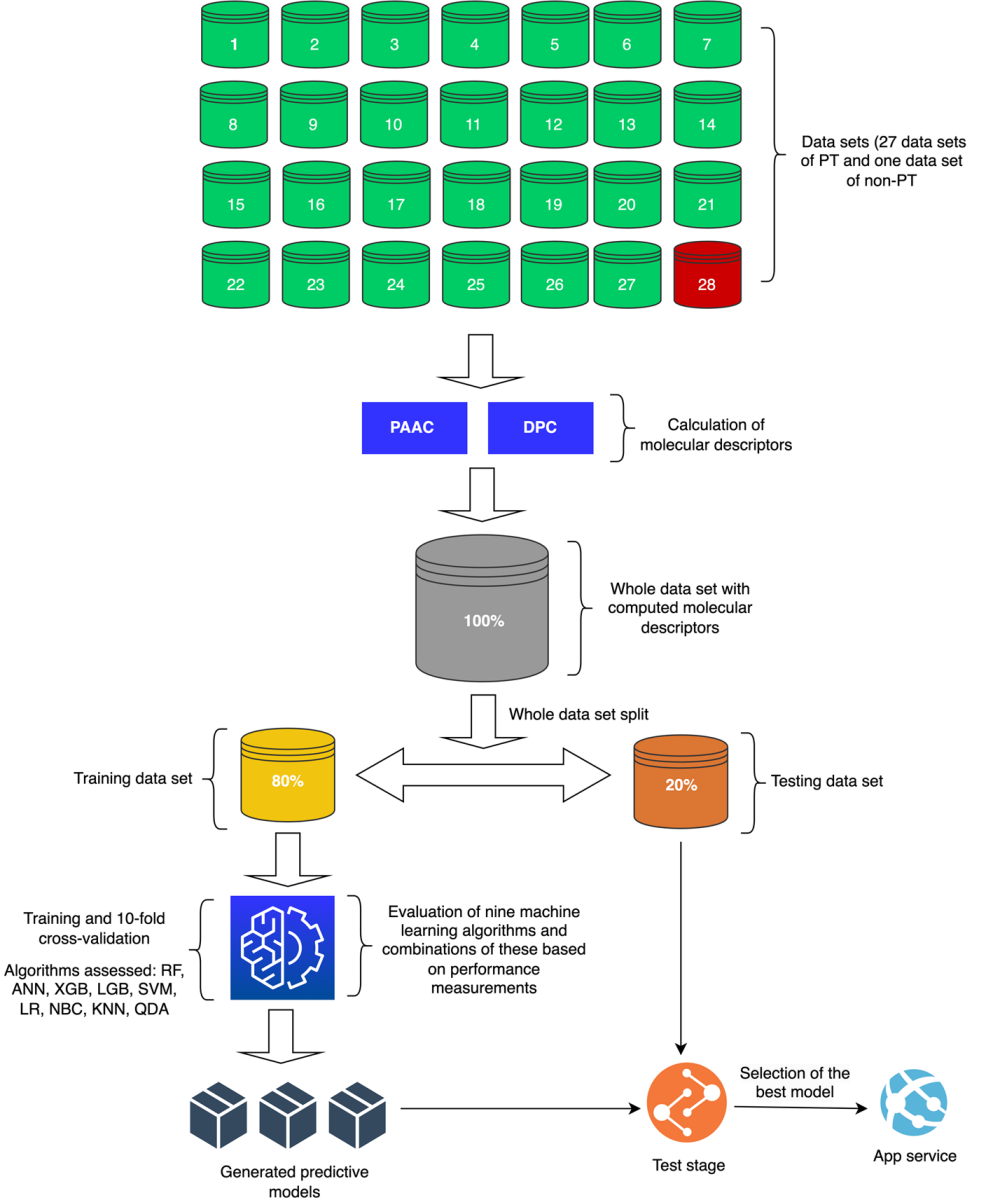
From the total dataset of amino acid sequences corresponding to different types of protein toxins with different modes of action in the cell (n = 27) and non-toxins (n = 1) randomly generated, the molecular descriptors PAAC and DPC were calculated. Subsequently, eight machine learning algorithms were evaluated, first on a training dataset (80%) which was subjected to tenfold cross-validation. Then, the generated models were evaluated on a test dataset (20%) (independent dataset). The final stage consisted of selecting the best predictive model for its incorporation into a web application called MultiToxPred 1.0

In this research, we also assessed the effectiveness of the predictive models using the area under the curve (AUC) of the receiver operating characteristic (ROC) plot. A modern web application was developed using the Python 3.11 programming language (https://www.python.org/) for making predictions of PT. The first version of the web application, named MultiToxPred 1.0, scores the outputs with a probability from 0 to 1. Figure 1 shows the working architecture used in this study.

## Results

The tenfold cross-validation analysis on the training data showed that the RF, XGBoost, and LightGBM algorithms displayed the best performance in the classification of PTs using the PAAC molecular descriptor (Table 1). On the other hand, when evaluating the DPC molecular descriptor, it was observed that LightGBM again showed good performance, as did the MLP and QDA algorithms (Table 2). The LR algorithm showed good performance with the use of DPC, however, low performance measures were obtained with PAAC and NB, with the latter algorithm having the worst performance with both evaluated descriptors (Tables 1 and 2). In the testing stage (Tables 3 and 4), in general, there was a consistent increase in the evaluated performance measures, which is indicative that the models are efficient at predicting PTs on independent data sets.

**Table 1:** Ten-fold cross-validation on the training dataset using the PAAC molecular descriptor

Algorithm	ACC	F1	PPV	TPR	AUC
RF	0.760	0.753	0.750	0.758	0.97
MLP	0.745	0.731	0.732	0.744	0.98
XGBoost	0.754	0.749	0.748	0.752	0.98
LightGBM	0.754	0.750	0.749	0.752	0.98
LR	0.530	0.504	0.501	0.529	0.93
NB	0.451	0.414	0.447	0.450	0.90
*k*-NN	0.753	0.735	0.743	0.752	0.95
QDA	0.700	0.673	0.671	0.699	0.95

**Table 2:** Ten-fold cross-validation on the training dataset using the DPC molecular descriptor

Algorithm	ACC	F1	PPV	TPR	AUC
RF	0.774	0.767	0.765	0.773	0.97
MLP	0.791	0.782	0.784	0.789	0.98
XGBoost	0.780	0.776	0.775	0.779	0.98
LightGBM	0.781	0.777	0.777	0.780	0.98
LR	0.792	0.777	0.779	0.791	0.98
NB	0.675	0.649	0.686	0.674	0.95
*k*-NN	0.749	0.727	0.744	0.748	0.95
QDA	0.806	0.791	0.810	0.805	0.92

**Table 3:** Performance on the testing dataset using the PAAC molecular descriptor

Algorithm	ACC	F1	PPV	TPR	AUC
RF	0.764	0.761	0.755	0.768	0.97
MLP	0.742	0.733	0.730	0.748	0.98
XGBoost	0.757	0.756	0.753	0.761	0.98
LightGBM	0.757	0.757	0.754	0.761	0.98
LR	0.525	0.506	0.497	0.532	0.94
NB	0.445	0.411	0.444	0.448	0.90
*k*-NN	0.753	0.741	0.751	0.759	0.96
QDA	0.701	0.678	0.674	0.704	0.95

**Table 4:** Performance on the testing dataset using the DPC molecular descriptor

Algorithm	ACC	F1	PPV	TPR	AUC
RF	0.779	0.777	0.773	0.783	0.97
MLP	0.794	0.791	0.787	0.799	0.98
XGBoost	0.784	0.784	0.782	0.788	0.99
LightGBM	0.787	0.788	0.787	0.791	0.99
LR	0.793	0.781	0.783	0.797	0.98
NB	0.667	0.645	0.682	0.670	0.95
*k*-NN	0.745	0.730	0.754	0.752	0.95
QDA	0.814	0.802	0.826	0.818	0.93

Considering the performance of the best algorithms in this study, both in the training and testing stages, we proceeded to evaluate the development of predictive models of PTs using an ensemble approach. In this direction, for the case of the PAAC molecular descriptor, an ensemble of RF and LightGBM was generated. For the DPC molecular descriptor, three ensembles were evaluated: MLP + LightGBM, MLP + QDA, and LightGBM + QDA. It is important to note that, regardless of the descriptor evaluated, the ensemble-based strategy allowed for better performance measures compared to the individual algorithms, both in the training and testing stages (Table 5).

**Table 5:** Ten-fold cross-validation on the training and testing datasets using the PAAC and DPC molecular descriptors via ensemble algorithms

Ensemble algorithms	ACC	F1	PPV	TPR	AUC
*PAAC*					
RF + LightGBM ^Training−CV^	0.789	0.781	0.779	0.788	0.98
RF + LightGBM ^Testing^	0.800	0.796	0.793	0.803	0.99
*DPC*					
MLP + LightGBM ^Training−CV^	0.813	0.801	0.801	0.812	0.99
MLP + LightGBM ^Testing^	0.816	0.806	0.799	0.820	0.99
MLP + QDA^Training−CV^	0.831	0.817	0.822	0.830	0.99
MLP + QDA^Testing^	0.844	0.837	0.843	0.847	0.99
LightGBM + QDA^Training−CV^	0.840*	0.827*	0.836*	0.840*	0.99*
LightGBM + QDA^Testing^	0.846*	0.838*	0.847*	0.849*	0.99*

CV: cross-validation, *: best performance measurements obtained

In the case of DPC, it was observed that these performance measures increased significantly, to a degree > 0.8, which indicates the robustness of this approach using this molecular descriptor and the algorithms used in the ensemble technique (Table 5). In consequence, these results demonstrate that our predictive strategy constitutes a robust approach for the prediction of PTs, taking into account the complexity of the study problem, which involves a high number of classes (27 in total). Of all the ensemble strategies evaluated, we noted that the resulting model from the LightGBM and QDA algorithms performed best during the cross-validation and testing phases (Table 5). In this direction, this model was selected for incorporation into a web application.

The web application developed in this study presents a modern and intuitive user interface, which allows carrying out PTs predictions. The results of the analyses can be downloaded in a csv file and/or can be selected and ranked in the application based on their respective probabilistic score, where scores greater than 0.5 indicate the probability that an unknown amino acid sequence introduced by the user corresponds to one of the 27 proposed classes (PT type and non-PT) in this work. The application, named MultiToxPred 1.0, is in its first version and is available for free use at https://www.biochemintelli.com/MultiToxPred-v1.

## Discussion

Currently, proteins and peptides (PT) are being extensively studied due to their great potential as therapeutic drugs in the treatment of various diseases, including immunological conditions, metabolic disorders, and neurodegenerative diseases, among others [1, 2, 60, 61]. The diversity in chemical nature and the complexity of PT structures, which are often derived from varied natural sources, make the study of these biomolecules, in most cases, a laborious and costly task. This is reflected in the numerous in vitro and in vivo experimental trials needed to confirm their effectiveness and safety [32]. On the other hand, machine learning techniques represent a robust alternative to rapidly and cost-effectively approach the identification of the functionality of peptides and proteins. These methods can predict the properties and behavior of PT based solely on their primary sequence, which can expedite the drug development process [33].

As mentioned above, numerous studies focusing on the prediction of PT behavior have been conducted. However, to date, no approach has been evaluated for predicting the specific mode of action of these biomolecules within the cell. It is well-documented, for example, that PTs from venomous animals target ion channels, which are in turn classified into several types based on the ions they transport [62, 63]. Predicting a more specific mode of action would not only determine whether a protein or peptide is a toxin but would also allow the elucidation of its modes of action within the cell. In some cases, it may even reveal its molecular target. Certainly, this would have a significant impact on the field of PT study. Considering all the aspects mentioned above, the motivation of this study was focused on an "out of the box" approach. The present study allowed the development of robust strategies that facilitate the prediction of PT in numerous classes, using multiple classification techniques, in contrast to state-of-the-art methods and tools that are based solely on binary classification (PT or non-PT).

Both descriptors used in this study (PAAC and DPC), are widely used in most of the works that apply machine learning techniques for the prediction of the biological functionality of peptides and proteins. In this work, we demonstrate that through the combined use of the LightGBM and QDA algorithms, the best performance measures are obtained with DPC (Table 3). The DPC molecular descriptor is a technique used in bioinformatics that is responsible for representing the properties of proteins or peptides. This descriptor is based on the idea that each dipeptide (a chain of two amino acids) has particular physicochemical properties and its frequency in the protein can provide significant information about its structure and function. In other words, DPC represents the frequency of each possible dipeptide in the total sequence of a protein, thus providing a global view of its composition and, potentially, its biological behavior. It is a tool widely used in the prediction of protein functionality, as it provides a general portrait of the molecular composition of the protein of interest [58]. The DPC has been assessed in various predictive toxin studies using machine learning techniques, proving its efficacy in this domain [40, 41, 43, 49]. This aligns, to a degree, with the findings of our study.

For the first time, we evaluated the development of a predictive model using an ensemble approach with LightGBM and QDA for PT predictions, which allowed us to obtain the best performance measurements (Table 5). The LightGBM is a gradient boosting-based machine learning algorithm that differs from other boosting algorithms in its ability to handle large data sets and its computational efficiency. It uses a leaf-based tree growth approach instead of the traditional depth-based growth, allowing you to focus on the regions of greatest loss and improving model accuracy. These features make LightGBM particularly useful for tasks that require high efficiency and precision [64]. The QDA is a statistical classification technique used in supervised learning. This method is based on Bayesian inference and assumes that each class in the dataset has its own covariance matrix [65]. Both algorithms have also been used in the classification of peptides and proteins, for example, LightGBM has been used in the prediction of anti-cancer peptides [66], protein structural class [67], protein–protein interactions [68], protein-ATP binding residues [69], and ion channels [70], among others. On the other hand, QDA has been used in the prediction of tumor T-cell antigens [71], antimicrobial peptides [72, 73], protein motifs [74], and protein subcellular location [75], among others.

Addressing the challenges inherent in predicting the specific mode of action of PTs in the cell using machine learning techniques will undoubtedly be an important focus for future research. One significant challenge is dealing with imbalanced data, as in many cases, the availability of labeled data for certain classes of PT is limited compared to others. Oversampling methods could be useful, and in this work, we demonstrate that by using SMOTE it is possible to obtain robust predictive models for predicting the molecular targets of PTs. As demonstrated in this study, the SMOTE technique has been used for the augmentation of amino acid sequence data [76], and it is considered the most used oversampling technique due to its fast and good results [77]. However, the exploration of other synthetic data generation techniques for protein and peptides, such as the use of adversarial neural networks [76, 78, 79], could be considered in future work to achieve the same purpose, which could significantly improve the performance of the predictive models.

We believe that this study serves as an initial springboard for the development of machine learning-based predictive tools to predict the specific functionalities of protein toxins. By leveraging sophisticated machine learning algorithms, it is possible to analyze vast amounts of biological data and obtain meaningful insights that would otherwise be too complex or time-consuming to obtain through traditional methods. In this direction, we believe that MultiToxPred 1.0 represents a novel tool that could be key for the study of PTs.

## Conclusions

For the first time, this study demonstrated that using a multiple classification approach aided with SMOTE, it is possible to predict the mode of action of a PT in the cell. Of all the machine learning algorithms evaluated, the best performance was observed with the combination of LightGBM and QDA using the DPC molecular descriptor. The model generated with these two combined algorithms was selected for incorporation into the MultiToxPred 1.0 web application, a free resource that facilitates PT predictions. These results highlight the power of machine learning techniques in predicting the functionality of PTs and suggest that MultiToxPred 1.0 may be an important tool in the discovery of these proteins as well as in the therapeutic area.

## Availability and requirements

Project name: MultiToxPred-v1.

Project home page: https://www.biochemintelli.com/MultiToxPred-v1

Operating system(s): Platform independent.

Programming language: Python 3.9

Other requirements: Python 3.7 or higher, scikit-learn, biopython, numpy, and pandas.

License: MIT License.

Any restrictions to use by non-academics: None.

### Supplementary Information

**Additional file 1.** Toxin class information.

**Additional file 2.** ROC curves in 10-fold cross-validation phase using DPC.

**Additional file 3.** ROC curves in testing phase using DPC.

**Additional file 4.** ROC curves in 10-fold cross-validation phase using PAAC.

**Additional file 5.** ROC curves in testing phase using PAAC.

**Additional file 6.** ROC curves in 10-fold cross-validation phase using PAAC and DPC.

**Additional file 7.** ROC curves in testing phase using PAAC and DPC.

**Additional file 8.** Figure legends S1–S6.

## Data Availability

All the amino acid sequences as well as the code used in this study are available in the GitHub repository: https://github.com/jfbldevs/MultiToxPred.
