# Supplementary material for: MultiToxPred 1.0: a novel comprehensive tool for predicting 27 classes of protein toxins using an ensemble machine learning approach
Source: BMC Bioinformatics. 2024 Apr 12;25:148. doi: 10.1186/s12859-024-05748-z (PMC11010298; doi:10.1186/s12859-024-05748-z)
Supplement: Supplementary file 1 — Additional file 1. Toxin class information. [file 12859_2024_5748_MOESM1_ESM.docx]

**Table S1** Classes of toxins along with the respective number of primary sequences identified in the literature and downloaded from UniProt.

| **Toxin class** | **Number** |
| --- | --- |
| Acetylcholine receptor inhibiting toxin | 493 |
| Blood coagulation cascade activating toxin | 107 |
| Blood coagulation cascade inhibiting toxin | 133 |
| Bradykinin receptor impairing toxin | 35 |
| Calcium-activated potassium channel impairing toxin | 72 |
| Cell adhesion impairing toxin | 228 |
| Chloride channel impairing toxin | 20 |
| Complement system impairing toxin | 27 |
| Dermonecrotic toxin | 216 |
| Enterotoxin | 101 |
| Fibrinogenolytic toxin | 102 |
| Fibrinolytic toxin | 41 |
| G-protein coupled acetylcholine receptor impairing toxin | 29 |
| G-protein coupled receptor impairing toxin | 228 |
| Hemorrhagic toxin | 62 |
| Hemostasis impairing toxin | 942 |
| Platelet aggregation activating toxin | 74 |
| Platelet aggregation inhibiting toxin | 350 |
| Potassium channel impairing toxin | 664 |
| Proton-gated sodium channel impairing toxin | 25 |
| Ryanodine-sensitive calcium-release channel impairing toxin | 27 |
| Target cell cytoplasm | 16 |
| Target cell membrane | 418 |
| Voltage-gated calcium channel impairing toxin | 247 |
| Voltage-gated chloride channel impairing toxin | 18 |
| Voltage-gated potassium channel impairing toxin | 508 |
| Voltage-gated sodium channel impairing toxin | 840 |
