# Supplementary material for: MultiToxPred 1.0: a novel comprehensive tool for predicting 27 classes of protein toxins using an ensemble machine learning approach
Source: BMC Bioinformatics. 2024 Apr 12;25:148. doi: 10.1186/s12859-024-05748-z (PMC11010298; doi:10.1186/s12859-024-05748-z)
Supplement: Supplementary file 8 — Additional file 8. Figure legends S1–S6. [file 12859_2024_5748_MOESM8_ESM.docx]

**S1** ROC curves were generated during the 10-fold cross-validation phase, using the DPC molecular descriptor, on the training data. (A) *k*-NN, (B) LightGBM, (C) LR, (D) MLP, (E) NB, (F) RF, (G) XGBoost, and (H) QDA.

**S2** ROC curves were generated during the testing phase, using the DPC molecular descriptor, on the test data. (A) k-NN, (B) LightGBM, (C) LR, (D) MLP, (E) NB, (F) RF, (G) XGBoost, and (H) QDA.

**S3** ROC curves were generated during the 10-fold cross-validation phase, using the PAAC molecular descriptor, on the training data. (A) k-NN, (B) LightGBM, (C) LR, (D) MLP, (E) NB, (F) RF, (G) XGBoost, and (H) QDA.

**S4** ROC curves were generated during the testing phase, using the PAAC molecular descriptor, on the test data. (A) k-NN, (B) LightGBM, (C) LR, (D) MLP, (E) NB, (F) RF, (G) XGBoost, and (H) QDA.

**S5** ROC curves were generated during the 10-fold cross-validation phase, using the PAAC and DPC molecular descriptors, on the training data. (A) LightGBM + QDA, (B) MLP + LightGBM, (C) MLP + QDA and (D) RF + LightGBM.

**S6** ROC curves were generated during the testing phase, using the PAAC and DPC molecular descriptors, on the training data. (A) LightGBM + QDA, (B) MLP + LightGBM, (C) MLP + QDA and (D) RF + LightGBM.
